# Frequent loss of expression without sequence mutations of the *DCC* gene in primary gastric cancer

**DOI:** 10.1054/bjoc.2001.1888

**Published:** 2001-07

**Authors:** K Sato, G Tamura, T Tsuchiya, Y Endoh, O Usuba, W Kimura, T Motoyama

**Affiliations:** Departments of Pathology, Surgery, Yamagata University School of Medicine, 2-2-2 Iida-nishi, Yamagata, 990-9585, Japan

**Keywords:** *DCC*, gastric cancer, mutation, methylation

## Abstract

Loss of heterozygosity (LOH) on chromosome 18q21 is frequently found in various human cancers, suggesting the presence of tumour suppressor gene(s) in this chromosomal region. *DCC* is the most likely target of LOH because loss or reduction of DCC expression has been found in many types of cancers. However, few reports have focused on sequence mutations of this gene. We investigated sequence mutations and expression of DCC in primary gastric cancers. We studied mutations in 25 of the 29 *DCC* exons by PCR-SSCP in 17 primary gastric cancers exhibiting LOH on 18q21. No mutations of *DCC* were found in any of the tumours, although 78% (47/60) of the primary tumours showed apparent loss or reduction of DCC expression by immunohistochemistry. Analysis of methylation status of *DCC* revealed that methylation frequently occurred in both primary tumours (75%; 45/60) and corresponding non-cancerous gastric mucosae (72%; 43/60). Methylated status of *DCC* was significantly correlated with the loss of DCC expression in primary tumours (*P*< 0.01). These results indicate that DCC is frequently silenced, probably by epigenetic mechanisms instead of sequence mutations in gastric cancer. © 2001 Cancer Research Campaign http://www.bjcancer.com


*British Journal of Cancer* (2001) **85**(2), 199–203
© 2001 Cancer Research Campaign
doi: 10.1054/ bjoc.2001.1888, available online at http://www.idealibrary.com on IDEAL®

http://www.bjcancer.com

### Frequent loss of expression without sequence mutations of the *DCC* gene in primary gastric cancer

**K Sato[1,2], G Tamura[1], T Tsuchiya[1], Y Endoh[1], O Usuba[2], W Kimura[2] and T Motoyama[1]**

Departments of [1]Pathology and [2]Surgery, Yamagata University School of Medicine, 2-2-2 Iida-nishi, Yamagata 990-9585, Japan

**Summary** Loss of heterozygosity (LOH) on chromosome 18q21 is frequently found in various human cancers, suggesting the presence of tumour suppressor gene(s) in this chromosomal region. *DCC* is the most likely target of LOH because loss or reduction of DCC expression has been found in many types of cancers. However, few reports have focused on sequence mutations of this gene. We investigated sequence mutations and expression of DCC in primary gastric cancers. We studied mutations in 25 of the 29 *DCC* exons by PCR-SSCP in 17 primary gastric cancers exhibiting LOH on 18q21. No mutations of *DCC* were found in any of the tumours, although 78% (47/60) of the primary tumours showed apparent loss or reduction of DCC expression by immunohistochemistry. Analysis of methylation status of *DCC* revealed that methylation frequently occurred in both primary tumours (75%; 45/60) and corresponding non-cancerous gastric mucosae (72%; 43/60). Methylated status of *DCC* was significantly correlated with the loss of DCC expression in primary tumours ($P < 0.01$). These results indicate that DCC is frequently silenced, probably by epigenetic mechanisms instead of sequence mutations in gastric cancer. © 2001 Cancer Research Campaign http://www.bjcancer.com

**Keywords**: *DCC*; gastric cancer; mutation; methylation

Studies have revealed a consistent set of genetic alterations, such as activation of proto-oncogenes and inactivation of tumour suppressor genes, in a variety of human malignancies. Because of the variety of histological types and mutagenetic substances, the molecular pathogenesis of gastric cancers is largely unknown. It is known, however, that there is some involvement of mutations in *p53* (Tamura et al, 1991) and *E (epithelial)-cadherin* genes (Tamura et al, 1996a), as well as microsatellite instability (MSI) due to mismatch repair deficiency (Tamura, 1995). Loss of heterozygosity (LOH) studies have suggested the presence of tumour suppressor genes on several chromosomal arms (Tamura et al, 1995, 1996b). However, the few genes that have been isolated from these regions, such as *APC* on 5q21 and *DPC4 (Smad4)* on 18q21, exhibit absent or infrequent mutations in gastric cancers (Maesawa et al, 1995; Nishizuka et al, 1997). LOH on chromosome 18q21 is frequently found in gastric cancers (Uchino et al, 1992; Tamura et al, 1996b; Nishizuka et al, 1998), and *DCC* has been postulated to be the major target. However, few reports have focused on *DCC* gene mutations and its mutational status is unknown in gastric cancer, probably because of the length and complexity of this gene (Fearon et al, 1990). *DPC4 (Smad4)*, another tumour suppressor gene on 18q21, exhibited frequent mutations accompanied by LOH in pancreatic cancers (Hahn et al, 1996), but no mutations have been found in gastric cancers (Nishizuka et al, 1997).

Aberrant DNA methylation of promoter CpG islands serves as an alternative mechanism to coding region mutation for the inactivation of tumour suppressor or tumour-related genes, including retinoblastoma (*RB*), von Hippel-Lindau (*VHL*), *p16, p15, hMLH1*, and *E-cadherin* (Herman et al, 1996, 1998; Graff et al, 1997; Stirzaker et al, 1997). Because gastric cancer displays the CpG island methylator phenotype (Toyota et al, 1999), and promoter hypermethylation of *p16, hMLH1*, and *E-cadherin* genes has been detected in gastric cancers (Fleisher et al, 1999; Toyota et al, 1999; Tamura et al, 2000), it is possible that *DCC* may also be affected by this epigenetic event.

We investigated sequence mutations, expression, and methylation status of *DCC* in primary gastric cancers. We found frequent loss of DCC expression in relation to hypermethylation but not in relation to sequence mutations.

### MATERIALS AND METHODS

### Primary gastric cancers

60 pairs of cancerous and non-cancerous tissues were surgically obtained from gastric cancer patients (13 differentiated and 17 undifferentiated carcinomas at the early stage, and 11 differentiated and 19 undifferentiated carcinomas at the advanced stage). These tissues were immediately frozen and stored at –80°C until analysis.

### Preparation of DNA

DNA was extracted from the 60 primary gastric cancers and their corresponding non-cancerous gastric mucosae with SepaGene (Sankojunyaku, Tokyo, Japan).

### Microsatellite analysis

LOH was examined using three polymorphic microsatellite markers, D18S474, D18S46 and DCC, obtained from MapPairs (Research Genetics, Huntsville, AL) on 18q21. PCR mix contained

*Received 18 December 2000*
*Revised 15 March 2001*
*Accepted 27 March 2001*

*Correspondence to:* G Tamura

1 × PCR buffer [15 mM Tris-HCl (pH 8.0), 50 mM KCl], 1.5 mM $MgCl_2$, deoxynucleotide triphosphates (each at 200 µM), 2.5 µCi of [α-$^{32}$P] dCTP (Amersham, Buckinghamshire, England), primers (1 µM each per reaction), 0.5 U AmpliTaq Gold™ DNA polymerase (PE Applied Biosystems, Foster City, CA) and genomic DNA (100 ng) in a final volume of 10 µl. Amplification was carried out in a GeneAmp PCR System 2400 (PE Applied Biosystems) for 35 cycles (15 s at 95°C, 15 s at appropriate annealing temperature, then 30 s at 72°C), followed by a final 7-min extension at 72°C. PCR products were diluted 1:10 in denaturing loading buffer [95% formamide, 10 mM EDTA (pH 8.0), 0.02% xylene cyanol FF, and 0.02% bromphenol blue], heated at 95°C for 5 min, placed on ice, and then 1.5 µl were subjected to electrophoresis. Gels for microsatellite analysis consisted of 6% polyacrylamide and 7 M urea. Gels were dried and exposed to Hyperfilm MP autoradiography film (Amersham) for 2–16 h.

### PCR-SSCP and sequencing

25 pairs of primers, including intron–exon boundaries, were used to amplify 25 of the 29 *DCC* exons, which have been described by Kong et al (1997). PCR conditions and products treatment for SSCP were the same as described for microsatellite analysis. Gels for SSCP analysis consisted of 6% polyacrylamide and 5% glycerol. Direct sequencing was performed using a small piece of the gel containing the shift band detected by SSCP. The gel was immersed in 50 µl of water, heated at 95°C, and then applied to PCR under the conditions described above for SSCP except that PCR was carried out in a volume of 50 µl. The PCR products were directly loaded onto nondenaturing 2% agarose gels and purified using QIA quick Gel Extraction Kit (QIAGEN, Tokyo, Japan). The purified PCR products were sequenced with the dRhodamine Terminator Cycle Sequencing Kit (PE Applied Biosystems). Gel electrophoresis, data collection and analysis were done with a Genetic Analyzer (model 310, PE Applied Biosystems).

### Methylation-specific PCR (MSP)

DNA methylation status was determined by MSP, as described previously (Herman et al, 1996). MSP distinguishes unmethylated from methylated alleles in a given gene based on sequence changes produced after bisulfite treatment of DNA, which converts unmethylated, but not methylated cytosines to uracil. Subsequently, PCR is performed using primers specific to either methylated or unmethylated DNA. Briefly, 1 µg of DNA was denatured by treatment with NaOH and modified by sodium bisulfite. DNA samples were then purified using Wizard DNA purification resin (Promega, Madison, WI), again treated with NaOH, precipitated with ethanol, and resuspended in water. Since the sequence of *DCC* promoter has not been available, we obtained the sequence of 5′ flanking region by direct sequencing and designed the following primers flanking the start codon. The primers used were 5′-CGTTGTTCGCGATTTTTGGTTTC-3′ (–41 to –19 bp from the start codon) and 5′-ACCGATTACTTAAAAATACGCG-3′ (71 to 92 bp from the start codon) for methylated (134 bp); and 5′-GTTGTTGTTGTTTGTGATTTTTGGTTTT-3′ (–46 to –19 bp from the start codon) and 5′-CCACTTACCAATTACTTAAAAATACACA-3′ (71 to 98 bp from the start codon) for the unmethylated DNA (145 bp) (Gen Bank accession No. M32292). The PCR amplified region for methylated and unmethylated alleles contained 8 CpG dinucleotides, including 3 or 4 CpGs at the primer annealing sites, respectively. PCR was performed under the same conditions described for microsatellite analysis except for the final volume of 20 µl. Sss-I methylase-treated DNA (New England Biolabs, Inc, Beverly, MA) and normal peripheral blood DNA served as positive and negative controls after bisulfite-modification, respectively. 10 µl samples of each PCR reaction product were directly loaded onto nondenaturing 6% polyacrylamide gels, stained with ethidium bromide, and visualized under UV illumination.

### Immunohistochemistry

Paraffin-embedded sections were evaluated immunohistochemically with a purified mouse anti-human DCC monoclonal antibody (clone G97–449, Pharmingen, San Diego, CA). Individual tissue sections of 3 µm were deparaffinized and heated in a 10 mM citric acid monophosphate buffer (pH 6) for 30 minutes in a 1.35-kW microwave oven at high power for antigen retrieval. The primary antibody was used at a dilution of 1:100. Sections were stained by the immunoperoxidase method with a streptavidin-biotin (SAB) complex system (Nichirei, Tokyo, Japan). Slides were counterstained with methylgreen. Immunoreactivity was judged as positive when at least 25% of tumour cells were immunoreactive with the DCC monoclonal antibody.

### Statistics

Statistical significance of difference was evaluated by Fisher's exact test with a criterion of $P < 0.05$.

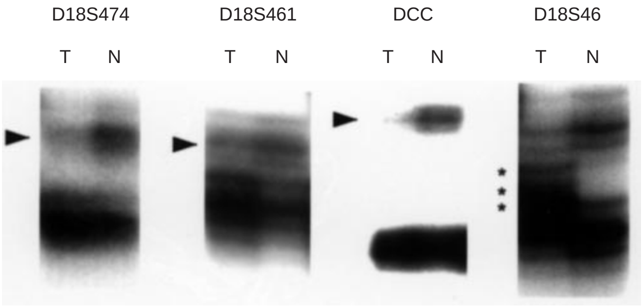

**Figure 1** Microsatellite analysis in primary gastric cancers. Apparent reduction in intensity of one of two bands in tumour DNA indicates LOH (arrowheads), and the presence of additional bands in tumour DNA, which are not seen in normal DNA, indicates MSI (asterisks). T, tumour DNA; N, corresponding gastric mucosa DNA

**Table 1** Histological type and LOH on 18q21

| Clinical stage and histological type | N* | LOH**(%) |
|---|---|---|
| Early-differentiated | 11 | 6 (55)[a] |
| Early-undifferentiated | 14 | 1 (7)[b] |
| Advanced-differentiated | 9 | 2 (22)[c] |
| Advanced-undifferentiated | 18 | 8 (44)[d] |
| Total | 52 | 17 (33) |

*, number of informative cases; **, number of cases exhibiting LOH on 18q21; [a]*vs.* [b], [b]*vs.* [d], $P < 0.05$; [b]*vs.*[a+c+d], $P < 0.015$.

*© 2001 Cancer Research Campaign*

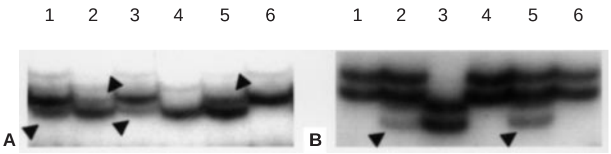

**Figure 2** PCR-SSCP analysis of exon 3 (**A**) and exon 19 (**B**) in *DCC*. Mobility shifts due to polymorphisms are clearly observed. One of polymorphic bands exhibits marked reduction in intensity due to LOH in lanes 1, 2, 3 and 5 of panel **A** and in lanes 2 and 5 of panel **B** (arrowheads)

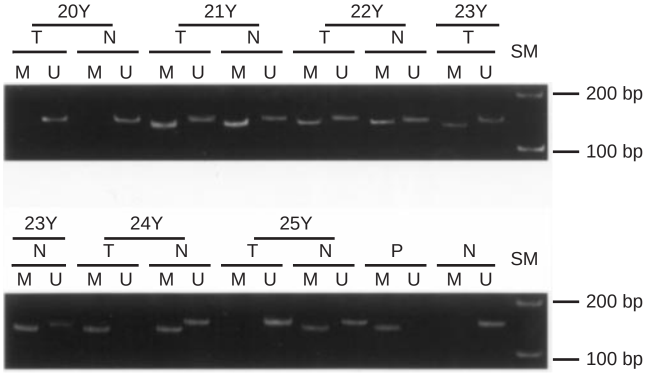

**Figure 3** MSP of 6 primary gastric cancers and their corresponding normal gastric mucosae. Methylated alleles are present in the primary tumour (T) of 21Y, 22Y, 23Y and 24Y, and in the normal mucosa (N) of 21Y, 22Y, 23Y, 24Y and 25Y. M, methylated allele; U, unmethylated allele; P, positive control; N, negative control; and SM, size marker

**Table 2** Methylation status and expression of DCC in primary gastric cancer

| Methylation status | | DCC expression | |
|---|---|---|---|
| | N* | Lost or reduced (%) | Expressed (%) |
| Methylated | 45 | 40 (89) | 5 (11)[a] |
| Unmethylated | 15 | 7 (47) | 8 (53)[b] |

*, number of tumours; [a]*vs.* [b], $P < 0.01$.

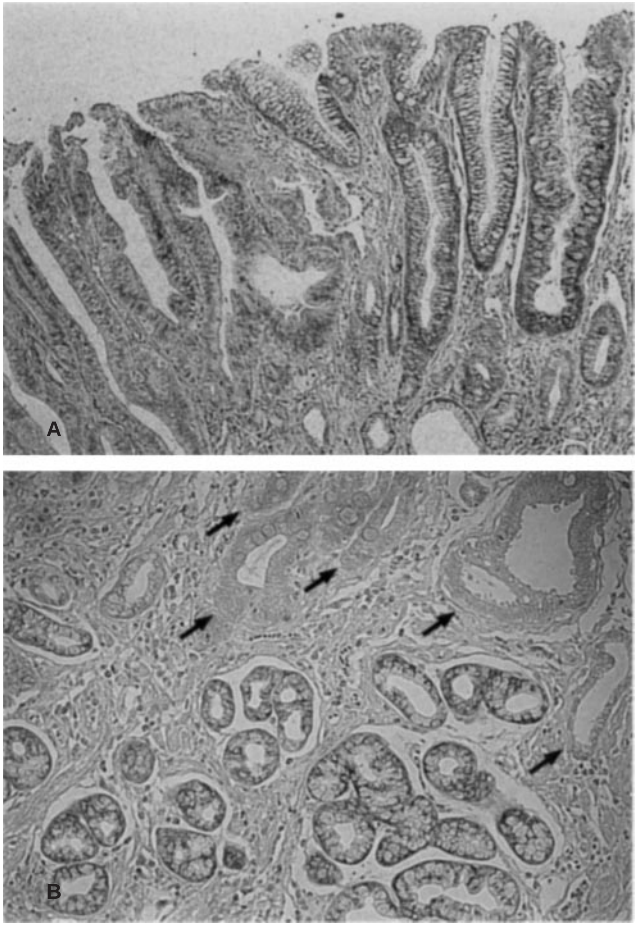

**Figure 4** Immunohistochemical analysis of DCC in primary gastric cancer and intestinal metaplasia. (**A**) Complete loss of DCC protein expression in primary gastric cancer (left half), and strong DCC protein expression in surrounding intestinal metaplasia (right half). (**B**) Reduced expression of DCC protein in intestinal metaplasia (arrows) relative to surrounding pyloric glands

### RESULTS

### LOH on 18q21

The frequencies of LOH at each microsatellite marker were 30% (7/23 informative cases) at D18S474, 29% (10/34) at D18S46 and 23% (5/22) at DCC, respectively. LOH on at least one of the 3 microsatellite markers was detected in 17 (33%) of 52 informative cases among 60 primary gastric cancers (Figure 1). The other 8 gastric cancers showed homozygosity for all markers. LOH is significantly less frequent in undifferentiated cancers at the early stage (7%; 1/14) than in others (42%; 16/38) ($P = 0.015$) (Table 1).

### *DCC* mutations

No somatic mutations were detected in 25 of 29 *DCC* exons evaluated in any of the 17 gastric cancers exhibiting LOH on 18q21. However, polymorphisms at codon 201 (CGA/GGA) in exon 3 (Figure 2A) and at codon 951 (TTT/TTG) in exon 19 (Figure 2B) were observed by PCR-SSCP. These polymorphisms have been reported by Kong et al (1997).

### Methylation status and expression of *DCC* in primary gastric cancers and corresponding normal gastric mucosae

45 (75%) of the 60 primary tumours and 43 (72%) of the 60 corresponding normal gastric mucosae showed the presence of methylated *DCC* alleles (Figure 3). This methylation in non-cancerous gastric mucosae may be an age-related phenomenon because gastric mucosa DNAs from autopsies of a 21-week-old fetus and a 16-year-old female did not exhibit these methylated alleles (data not shown). Methylated alleles were present in both the tumour and normal pairs in 32 (53%), only in the tumour in 13 (22%), only in the normal mucosa in 11 (18%), and in neither of the samples in the remaining 4 (7%) of the 60 cases.

Normal fundic and pyloric glands showed positive immunoreactivity against the DCC monoclonal antibody similar to a previous report using the same antibody (Yoshida et al, 1998). In primary tumors, 47 (78%) of 60 showed negative immunoreactivity for DCC (Figure 4A). However, immunoreactivity in intestinal metaplasia varied in each case, or even from area to area within the same case (Figure 4B). Diminished DCC immunoreactivity was observed in 40 (89%) of the 45 methylated and 7 (47%) of the 15 unmethylated tumours ($P < 0.01$) (Table 2).

© 2001 Cancer Research Campaign

### DISCUSSION

We have demonstrated that *DCC* is not structurally altered in primary gastric cancers, although it is possible that some of our cases may carry mutations in other exons than in those we have studied. However, 78% (47/60) of the primary tumours exhibited apparent loss of or reduction in DCC expression. This is the first report investigating mutations of *DCC* in gastric cancer. Previous reports have addressed infrequent mutations of this gene in colorectal (Fearon et al, 1990; Cho et al, 1994), oesophageal (Miyake et al, 1994) and neurogenic malignancies (Kong et al, 1997). All of those tumour types frequently lost DCC expression and/or exhibited LOH at 18q21 (Miyake et al, 1994; Kong et al, 1997; Schmitt et al, 1998). In gastric cancer, frequent LOH on 18q has been reported in differentiated cancers but was infrequent in diffuse infiltrative lesions (Uchino et al, 1992; Yoshida et al, 1998). In the present study, LOH on 18q21 was infrequent in early undifferentiated cancers, but frequent in both advanced undifferentiated and differentiated cancers. Because a significant proportion of differentiated gastric cancers progress to undifferentiated ones (Ikeda et al, 1994; Endoh et al, 1999), advanced gastric cancers of both histological types share common genetic alterations. Our present results on LOH at 18q21 support the hypothesis that the genetic pathways involved in differentiated and undifferentiated gastric cancers are distinct at their early stages. Similarly, infrequent mutations of *p53* in early undifferentiated gastric cancers have been reported (Wu et al, 1997).

Loss of or reduction in *DCC* expression has been reported to occur in 40% (Kataoka et al, 1995) or 52% (Yoshida et al, 1998) of gastric cancers. The pronounced rates of diminished DCC expression in our study may have resulted from the method we employed for the detection of DCC expression in primary tumours. There were no significant differences of incidences observed between loss of DCC expression and histological type or stage in our study.

Hypermethylation of promoter regions near transcriptional start sites correlated well with the loss or reduction observed in gene expression (Baylin et al, 1998). Because the promoter sequence of *DCC* has not yet been identified, we designed primers flanking the start codon for MSP. Methylation originates in either flanking region and spreads to include the promoter CpG islands near the transcriptional start site (Graff et al, 1997). In the present study, diminished DCC expression was observed not only in methylated but also in unmethylated primary tumours. In addition, DCC was expressed in some primary tumours exhibiting methylated alleles. These discrepancies between methylation status and DCC expression might have resulted from differences in methylation status between the heart of promoter CpG islands and the region we studied. Methylation of only a small region of the *hMLH1* promoter has been found to be sufficient to block expression (Deng et al, 1999). It is also possible that other mechanisms which interfere with DCC expression might be involved. Thus, methylation of *DCC* at the region studied did not always silence gene expression. There is no evidence that shows this methylated portion is involved in transcriptional activity, however, the significant correlation between *DCC* methylation and loss of gene expression in primary gastric cancers may suggest that this epigenetic phenomenon plays a role in frequent loss of DCC expression.

Methylation was also frequently observed in corresponding non-cancerous gastric mucosae, although no methylated *DCC* alleles were present in the stomach of a fetus and a 16-year-old female (data not shown). For the *ER* and *IGF2* genes, methylation begins in the normal colonic mucosa as an age-related event and progresses to hypermethylation in cancer (Ahuja et al, 1998). The protection from de novo methylation by Sp1 elements may be lost during aging (Ahuja et al, 1998). In a separate study, we found that an *APC* promoter was usually methylated in the normal gastric mucosae of elderly subjects (Tsuchiya et al, 2000) but was not methylated in the fetus and the young female. In addition, the *APC* promoter was not methylated in normal colonic mucosa (Hiltunen et al, 1997). Therefore, age-related methylation might be modulated by variable agents in a tissue-specific manner. In the present study, we did not find a significant correlation between patient's age and *DCC* methylation status in normal gastric mucosae, perhaps because most of the patients were older than 50 years of age (range, 30–85; average, 65). Recently, frequent methylation of the *hMLH1* promoter in colonic mucosae as well as in colon cancers has been reported (Herman et al, 1998). Hypermethylation of *hMLH1* in colon cancers relative to normal colonic mucosae correlated well with functional loss of hMLH1 assessed by the presence of microsatellite instability (Kuismanen et al, 1999). We found variable immunoreactivity for DCC in intestinal metaplasia, which is commonly observed in the elderly Japanese population. We speculate that methylation of *DCC* might initially occur in intestinal metaplasia, and then progress to cancer, although the relationship between methylation status and immunoreactivity for DCC in intestinal metaplasia remains to be determined. Further studies on intestinal metaplasia using microdissection will resolve this issue.

Because of the small number of tumours exhibiting LOH on 18q21, we failed to show significant correlation between methylation and expression of DCC in primary tumours exhibiting LOH on 18q21, however, DCC expression was diminished more frequently in methylated tumours than in unmethylated tumours among 17 tumours exhibiting LOH on 18q21. *p16* hypermethylation with concordant LOH on 9p21 has been reported in non-small cell lung cancer (Kohno and Yokota, 1999). Therefore, it is possible that LOH on 18q21 accompanied by methylation may silence DCC expression in gastric cancers. It is also possible that both alleles were affected by methylation in tumours retaining both alleles.

In conclusion, *DCC* is not mutated in gastric cancers, and frequent loss of DCC expression might therefore result from an epigenetic phenomenon.

### ACKNOWLEDGEMENTS

This work was supported by a Grant-in-Aid for Cancer Research (No. 10–3) from the Ministry of Health and Welfare, and by a Grant-in-Aid (12670154) from the Ministry of Education, Science, Sports and Culture of Japan.

### REFERENCES

Ahuja N, Li Q, Mohan AL, Baylin SB and Issa JP (1998) Aging and DNA methylation in colorectal mucosa and cancer. *Cancer Res* **58**: 5489–5494

Baylin SB, Herman JG, Graff JR, Vertino PM and Issa JP (1998) Alterations in DNA methylation: a fundamental aspect of neoplasia. *Adv Cancer Res* **72**: 141–196

Cho KR, Oliner JD, Simons JW, Hedrick L, Fearon ER, Preisinger AC, Hedge P, Silverman GA and Vogelstein B (1994) The *DCC* gene: structural analysis and mutations in colorectal carcinomas. *Genomics* **19**: 525–531

Deng G, Chen A, Hong J, Chae HS and Kim YS (1999) Methylation of CpG in a small region of the *hMLH1* promoter invariably correlates with the absence of gene expression. *Cancer Res* **59**: 2029–2033

*© 2001 Cancer Research Campaign*

Endoh Y, Tamura G, Watanabe H, Ajioka Y and Motoyama T (1999) The common 18-base pair deletion at codons 418–423 of the *E-cadherin* gene in differentiated-type adenocarcinomas and intramucosal precancerous lesions of the stomach with the features of gastric foveolar epithelium. *J Pathol* **189**: 201–206

Fearon ER, Cho KR, Nigro JM, Kern SE, Simons JW, Ruppert JM, Hamilton SR, Preisinger AC, Thomas G and Kinzler KW et al. (1990) Identification of a chromosome 18q gene that is altered in colorectal cancers. *Science* **247**: 49–56

Fleisher AS, Esteller M, Wang S, Tamura G, Suzuki H, Yin J, Zou TT, Abraham JM, Kong D, Smolinski KN, Shi YQ, Rhyu MG, Powell SM, James SP, Wilson KT, Herman JG and Meltzer SJ (1999) Hypermethylation of the *hMLH1* gene promoter in human gastric cancers with microsatellite instability. *Cancer Res* **59**: 1090–1095

Graff JR, Herman JG, Myohanen S, Baylin SB and Vertino PM (1997) Mapping patterns of CpG island methylation in normal and neoplastic cells implicates both upstream and downstream regions in de novo methylation. *J Biol Chem* **272**: 22322–22329

Hahn SA, Schutte M, Hoque AT, Moskaluk CA, da Costa LT, Rozenblum E, Weinstein CL, Fischer A, Yeo CJ, Hruban RH and Kern SE (1996) *DPC4*, a candidate tumor suppressor gene at human chromosome 18q21.1. *Science* **271**: 350–353

Herman JG, Graff JR, Myohanen S, Nelkin BD and Baylin SB (1996) Methylation-specific PCR: a novel PCR assay for methylation status of CpG islands. *Proc Natl Acad Sci U S A* **93**: 9821–9826

Herman JG, Umar A, Polyak K, Graff JR, Ahuja N, Issa JP, Markowitz S, Willson JK, Hamilton SR, Kinzler KW, Kane MF, Kolodner RD, Vogelstein B, Kunkel TA and Baylin SB (1998) Incidence and functional consequences of *hMLH1* promoter hypermethylation in colorectal carcinoma. *Proc Natl Acad Sci U S A* **95**: 6870–6875

Hiltunen MO, Alhonen L, Koistinaho J, Myohanen S, Paakkonen M, Marin S, Kosma VM and Janne J (1997) Hypermethylation of the *APC* (adenomatous polyposis coli) gene promoter region in human colorectal carcinoma. *Int J Cancer* **70**: 644–648

Ikeda Y, Mori M, Kamakura T, Haraguchi Y, Saku M and Sugimachi K (1994) Increased incidence of undifferentiated type of gastric cancer with tumor progression in 912 patients with early gastric cancer and 1245 with advanced gastric cancer. *Cancer* **73**: 2459–2463

Kataoka M, Okabayashi T and Orita K (1995) Decreased expression of *DCC* mRNA in gastric and colorectal cancer. *Surg Today* **25**: 1001–1007

Kohno T and Yokota J (1999) How many tumor suppressor genes are involved in human lung carcinogenesis? *Carcinogenesis* **20**: 1403–1410

Kong XT, Choi SH, Inoue A, Xu F, Chen T, Takita J, Yokota J, Bessho F, Yanagisawa M, Hanada R, Yamamoto K and Hayashi Y (1997) Expression and mutational analysis of the *DCC*, *DPC4*, and *MADR2/JV18-1* genes in neuroblastoma. *Cancer Res* **57**: 3772–3778

Kuismanen SA, Holmberg MT, Salovaara R, Schweizer P, Aaltonen LA, de La Chapelle A, Nystrom-Lahti M and Peltomaki P (1999) Epigenetic phenotypes distinguish microsatellite-stable and -unstable colorectal cancers. *Proc Natl Acad Sci U S A* **96**: 12661–12666

Maesawa C, Tamura G, Suzuki Y, Ogasawara S, Sakata K, Kashiwaba M and Satodate R (1995) The sequential accumulation of genetic alterations characteristic of the colorectal adenoma-carcinoma sequence does not occur between gastric adenoma and adenocarcinoma. *J Pathol* **176**: 249–258

Miyake S, Nagai K, Yoshino K, Oto M, Endo M and Yuasa Y (1994) Point mutations and allelic deletion of tumor suppressor gene *DCC* in human esophageal squamous cell carcinomas and their relation to metastasis. *Cancer Res* **54**: 3007–3010

Nishizuka S, Tamura G, Maesawa C, Sakata K, Suzuki Y, Iwaya T, Terashima M, Saito K and Satodate R (1997) Analysis of the *DPC4* gene in gastric carcinoma. *Jpn J Cancer Res* **88**: 335–339

Nishizuka S, Tamura G, Terashima M and Satodate R (1998) Loss of heterozygosity during the development and progression of differentiated adenocarcinoma of the stomach. *J Pathol* **185**: 38–43

Schmitt CA, Thaler KR, Wittig BM, Kaulen H, Meyer zum Buschenfelde KH and Dippold WG (1998) Detection of the *DCC* gene product in normal and malignant colorectal tissues and its relation to a codon 201 mutation. *Br J Cancer* **77**: 588–594

Stirzaker C, Millar DS, Paul CL, Warnecke PM, Harrison J, Vincent PC, Frommer M and Clark SJ (1997) Extensive DNA methylation spanning the Rb promoter in retinoblastoma tumors. *Cancer Res* **57**: 2229–2237

Tamura G (1996) Molecular pathogenesis of adenoma and differentiated adenocarcinoma of the stomach. *Pathol Int* **46:** 834–841

Tamura G, Kihana T, Nomura K, Terada M, Sugimura T and Hirohashi S (1991) Detection of frequent *p53* gene mutations in primary gastric cancer by cell sorting and polymerase chain reaction single-strand conformation polymorphism analysis. *Cancer Res* **51**: 3056–3058

Tamura G, Sakata K, Maesawa C, Suzuki Y, Terashima M, Satoh K, Sekiyama S, Suzuki A, Eda Y and Satodate R (1995) Microsatellite alterations in adenoma and differentiated adenocarcinoma of the stomach. *Cancer Res* **55**: 1933–1936

Tamura G, Sakata K, Nishizuka S, Maesawa C, Suzuki Y, Iwaya T, Terashima M, Saito K and Satodate R (1996a) Inactivation of the *E-cadherin* gene in primary gastric carcinomas and gastric carcinoma cell lines. *Jpn J Cancer Res* **87**: 1153–1159

Tamura G, Sakata K, Nishizuka S, Maesawa C, Suzuki Y, Terashima M, Eda Y and Satodate R (1996b) Allelotype of adenoma and differentiated adenocarcinoma of the stomach. *J Pathol* **180**: 371–377

Tamura G, Yin J, Wang S, Fleisher AS, Zou T, Abraham JM, Kong D, Smolinski KN, Wilson KT, James SP, Silverberg SG, Nishizuka S, Terashima M, Motoyama T and Meltzer SJ (2000) *E-Cadherin* gene promoter hypermethylation in primary human gastric carcinomas. *J Natl Cancer Inst* **92**: 569–573

Toyota M, Ahuja N, Suzuki H, Itoh F, Ohe-Toyota M, Imai K, Baylin SB and Issa JP (1999) Aberrant methylation in gastric cancer associated with the CpG island methylator phenotype. *Cancer Res* **59**: 5438–5442

Tsuchiya T, Tamura G, Sato K, Endoh Y, Sakata K, Jin Z, Motoyama T, Usuba O, Kimura W, Nishizuka S, Yin J, Fleisher AS, Zou T, Kong D and Meltzer SJ (2000) Distinct methylation patterns of two *APC* gene promoters in normal and cancerous gastric epithelia. *Oncogene* **19**: 3642–3646

Uchino S, Tsuda H, Noguchi M, Yokota J, Terada M, Saito T, Kobayashi M, Sugimura T and Hirohashi S (1992) Frequent loss of heterozygosity at the *DCC* locus in gastric cancer. *Cancer Res* **52**: 3099–3102

Wu MS, Shun CT, Wang HP, Sheu JC, Lee WJ, Wang TH and Lin JT (1997) Genetic alterations in gastric cancer: relation to histological subtypes, tumor stage, and Helicobacter pylori infection. *Gastroenterology* **112**: 1457–1465

Yoshida Y, Itoh F, Endo T, Hinoda Y and Imai K (1998) Decreased *DCC* mRNA expression in human gastric cancers is clinicopathologically significant. *Int J Cancer* **79**: 634–639

*© 2001 Cancer Research Campaign*
